# The Medical Genome Initiative: moving whole-genome sequencing for rare disease diagnosis to the clinic

**DOI:** 10.1186/s13073-020-00748-z

**Published:** 2020-05-27

**Authors:** Christian R. Marshall, David Bick, John W. Belmont, Stacie L. Taylor, Euan Ashley, David Dimmock, Vaidehi Jobanputra, Hutton M. Kearney, Shashikant Kulkarni, Heidi Rehm, on behalf of the Medical Genome Initiative

**Affiliations:** 1 Genome Diagnostics, The Hospital for Sick Children, Toronto, ON Canada; 2 HudsonAlpha Institute for Biotechnology, Huntsville, AL USA; 3 Illumina Inc., San Diego, CA USA; 4 Stanford Medicine Clinical Genomics Program, Stanford Health Care, Stanford, CA USA; 5 Rady Children’s Institute for Genomic Medicine, San Diego, CA USA; 6 New York Genome Center, New York, NY USA; 7 Mayo Clinic, Rochester, MN USA; 8 Baylor College of Medicine and Baylor Genetics, Houston, TX USA; 9 Broad Institute of MIT and Harvard, Cambridge, MA USA

**Keywords:** Clinical whole-genome sequencing, Diagnostics, Standards, Rare genetic disease

## Abstract

Clinical whole-genome sequencing (WGS) offers clear diagnostic benefits for patients with rare disease. However, there are barriers to its widespread adoption, including a lack of standards for clinical practice. The Medical Genome Initiative consortium was formed to provide practical guidance and support the development of standards for the use of clinical WGS.

## Background

Rare diseases affect more than 350 million people globally and collectively represent a particularly significant source of morbidity and mortality [1]. Many have an underlying genetic component as demonstrated by a recent review of > 3800 rare diseases listed by Orphanet, which showed that approximately 80% are either exclusively genetic or have genetic subtypes [2]. Patients with rare diseases commonly experience multiyear diagnostic evaluations and receive multiple misdiagnoses during that time [1]. Thus, establishing a precise molecular diagnosis can reduce costs by ending this diagnostic odyssey and, in many cases, aiding in medical management [3, 4].

The advent of next-generation sequencing technology has been transformative in the molecular diagnosis of rare disease by allowing comprehensive analysis of patient genomes. Clinical whole-genome sequencing (WGS) can detect a broad range of pathogenic allele types and is emerging as an effective first-tier test for cases in which physicians are faced with a high degree of diagnostic uncertainty [5]. Thus, clinical WGS in rare disease has the potential to deliver precise molecular diagnoses, enable changes in medical management, and eliminate the burden of the unknown that weighs on patients and their families [3, 4, 6].

Despite all this potential, the majority of individuals undergoing WGS to date have been tested through research protocols. There are currently several obstacles in transitioning WGS testing from the research setting into clinical practice. For laboratories, in addition to the significant capital cost required for set up, the initial steps of establishing a technically challenging test and becoming proficient in its interpretation and reporting can be overwhelming. For clinicians, education around WGS (i.e., the test itself, patient selection, clinical utility, reimbursement practices) and the value of a diagnosis to patients and families represent barriers to widespread adoption. Importantly, an additional significant barrier to widespread adoption is a general lack of guidance and standards for clinical implementation.

## The importance of standards

**Fig. 1:**
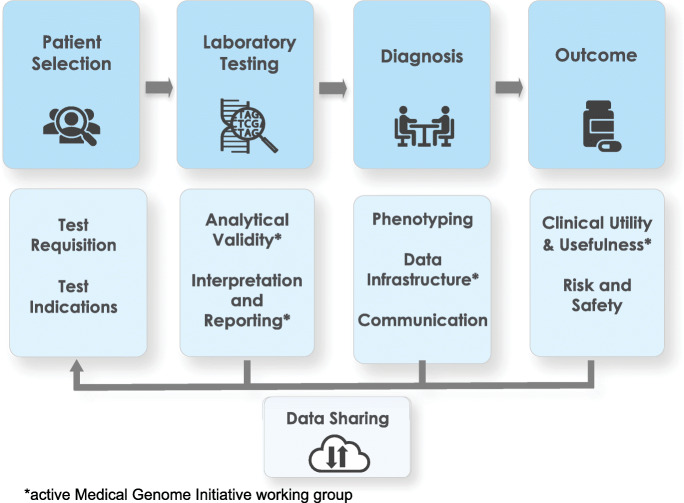
Key components of WGS supporting the core steps of genetic disease diagnosis. The top row of boxes represents the core steps involved in genetic disease diagnosis using clinical WGS. The second row highlights the key topics that are both associated with each step and where the Medical Genome Initiative observed limited or nonexistent clinical standards relevant to each topic

The definition and adoption of common international standards are essential for the transformation of WGS from a powerful research tool into a safe and effective diagnostic test. An important first step requires early adopters to share their experiences, compare operating procedures, and define areas throughout the testing process where there is clear consensus (Fig. 1). For example, to establish a consensus for clinical WGS test definition, stakeholders, such as clinical laboratory directors, physicians, health system administrators, and payers require standards so there are shared expectations of performance and quality. Standards also guide clinical research to better understand patient outcomes, unanticipated risks, and cost-effectiveness.

Developing standards for clinical WGS is particularly challenging due to the broad range of clinical settings, test indications, and rapidly evolving technical improvements. The Medical Genome Initiative was formed [7] in recognition of these challenges and the need for best practice recommendations to guide the implementation of WGS for clinical care.

## The Medical Genome Initiative

The Medical Genome Initiative is a consortium comprised of leading North American healthcare and research organizations with significant interest and experience in evaluating human genomes for the genetic basis of disease. Although chromosomal microarray analysis (CMA) and whole-exome sequencing (WES) are currently indicated as first-tier tests for many rare genetic diseases, the collective experience of Medical Genome Initiative members (spans 10 years and includes performing 33,000 WES and 220,000 CMA analyses) leads us to believe that WGS is ready to take over as the first-line test for this patient population. As a group, Medical Genome Initiative members have sequenced over 70,000 clinical grade genomes with 5000 of these being fully clinically interpreted and reported. The initiative is focused on publishing recommended laboratory and clinical best practices for the implementation of clinical WGS, along with clinical research frameworks to demonstrate the value clinical WGS brings to healthcare.

The major components of the whole-genome testing workflow are illustrated in Fig. 1. It begins with a patient with a suspected genetic disorder, followed by clinical laboratory WGS testing, then diagnostic confirmation through clinical correlation, and finally, the assessment of patient outcome. After critical evaluation and discussion of these components, the Medical Genome Initiative identified several key topics where standards relevant to clinical WGS were missing or limited and where practical guidance would be beneficial. The themes of analytical validity, clinical utility, data infrastructure, and test interpretation and reporting emerged as areas where this guidance was most needed. Working groups were formed to discuss current institutional practices and to identify areas of consensus across sites as well as areas where consensus was lacking.

## Facilitating the development of standards

Clinical WGS is a technically challenging service to establish and maintain. Although regulatory bodies (e.g., College of American Pathologists (CAP), Food and Drug Administration (FDA)) and professional societies (e.g., Association for Molecular Pathology (AMP), The American College of Medical Genetics and Genomics (ACMG)) have published broad guidelines that can be applied to clinical WGS setup [8–10], they do not address or offer guidance on the unique challenges of WGS which opens the door to individual interpretation and hinders standardization efforts. Similarly, other organizations like The Global Alliance for Genomics and Health (GA4GH) have collaborated with the National Institute of Standards and Technology to develop useful technical infrastructure and tools that can be applied broadly to analytical test validation but not specifically for clinical WGS. Thus, there is no standard or consensus for what constitutes a clinical WGS test nor what performance metric thresholds must be met. This ambiguity is a major challenge for clinical laboratory directors who are working to establish clinical WGS as a diagnostic test in their laboratories, and for healthcare payers deciding which laboratories are qualified to perform them. The Analytical Validity working group is focused on reducing this ambiguity by developing best practice recommendations for the analytical validation of clinical WGS in the context of germline disease diagnosis. Importantly, these efforts include defining analytical metrics and thresholds for clinical WGS that, at a minimum, show no loss in performance compared to WES and CMA. These metrics are poised to enable WGS performance to exceed other genetic testing modalities in sensitivity, specificity, and breadth of variant detection.

In most areas of clinical research, the effectiveness of an intervention can be easily tied to a predefined health outcome. In contrast, generating and evaluating evidence of clinical utility for WGS is complex and is further compounded by the diversity of rare diseases where WGS is expected to be relevant. The Clinical Utility and Usefulness Measures working group is focused on developing best practice recommendations for measuring utility as it relates to the diagnostic use of WGS. Building upon existing guidance and definitions of utility from organizations such as the Evaluation of Genomic Applications in Practice and Prevention working group and the ACMG, the group is developing a measurement toolkit that offers resources and practical guidance with an emphasis on objective and validated measures for the evaluation and generation of evidence related to clinical utility. Investigators, policy advisory bodies, payers, and healthcare systems committed to providing value-based care will be able to use the toolkit as a resource to guide empirical studies of utility and evaluations of the impact of WGS on healthcare outcomes.

Clinical WGS at the scale required for widespread healthcare system adoption presents many operational challenges for institutions. Understanding optimal genomic data infrastructure and regulatory compliance requirements are examples of these challenges. Both GA4GH and the International Organization for Standardization have provided guidance and standards for the storage and sharing of genomic health data. However, guidance and recommendations for what infrastructure is needed to set up clinical WGS are lacking due to the rapid pace at which the field is evolving. The Clinical Data Infrastructure working group is focused on describing current solutions and developing best practice recommendations for the storage and management of the extraordinary volume of sequence and health data generated by clinical WGS. The working group aims to identify and provide resources that address the unique challenges of data management infrastructure to support optimal data usage and sharing within a clinical diagnostic laboratory setting.

There are additional components of clinical WGS, such as tailoring analyses for specific patient indications, and determining optimal approaches to test interpretation and reporting, that would benefit from practical guidance from field expertise. Test interpretation and reporting for a clinical WGS test, in particular, are challenging due to the depth and breadth of genetic variation that is detectable. Due to the comprehensive nature of the test, the laboratory must prioritize detection of all variants relevant to the clinical phenotype while minimizing the return of highly uncertain or clinically irrelevant results. There is currently very little guidance or consensus from the ACMG or AMP that is specific to WGS on how best to accomplish this, though a multitude of valid and justifiable strategies exist. The Interpretation and Reporting working group is focused on developing best practice recommendations for the comprehensive identification of genomic variants most likely relevant to the patient’s phenotype. Further, the goal of the group is to promote best practices in reporting approaches for laboratories by discussing how best to differentiate primary results from secondary ones and incidental findings and how to manage the return of uncertain results.

## Future directions

In the future, the Medical Genome Initiative will develop best practice recommendations for the remaining key areas illustrated in Fig. 1, including guidance for health care providers related to patient selection, communication of results between the laboratory, health care providers, and patients, as well as regarding safety issues around the implementation of clinical WGS. It will continue to use a consensus-based approach to provide guidance and practical recommendations in areas that will support progress in the use of WGS for the diagnosis of patients with suspected genetic disease.

## Data Availability

Not applicable.

## Abbreviations

AMP: Association for Molecular Pathology
ACMG: The American College of Medical Genetics and Genomics
GA4GH: Global Alliance for Genomics and Health
WES: Whole-exome sequencing
WGS: Whole-genome sequencing
